# Supplementary material for: Exploring Alternative Pathways to Target Bacterial Type II Topoisomerases Using NBTI Antibacterials: Beyond Halogen-Bonding Interactions
Source: Antibiotics (Basel). 2023 May 18;12(5):930. doi: 10.3390/antibiotics12050930 (PMC10215804; doi:10.3390/antibiotics12050930)
Supplement: Supplementary file 1 [file antibiotics-12-00930-s001.zip › antibiotics-2337766-supplementary.pdf]

Supporting Information

Exploring alternative pathways to target the bacterial type II topoisomerases by NBTI antibacterials: beyond the halogen-bonding interactions

Maja Kokot [1,2], Doroteja Novak [2], Irena Zdovc [3], Marko Anderluh [2], Martina Hrast [2,*], Nikola Minovski [1,*]

*Corresponding authors: Tel. +386 1 4769 674 e-mail: martina.hrast@ffa.uni-lj.si, Tel. +386 1 4760 383, e-mail: nikola.minovski@ki.si

[1] Theory Department, Laboratory for Cheminformatics, National Institute of Chemistry, Hajdrihova 19, 1001 Ljubljana, Slovenia

[2] Department of Pharmaceutical Chemistry, Faculty of Pharmacy, University of Ljubljana, Aškerčeva cesta 7, 1000 Ljubljana, Slovenia

[3] Institute of Microbiology and Parasitology, Veterinary Faculty, University of Ljubljana, Gerbičeva 60, 1000 Ljubljana, Slovenia

Docking parameters

Table S1. Settings and parameters of the GOLD genetic algorithm

Setting/parameter	Value
Population size	100
Selection pressure	1.1
Number of operations	100,00
Number of islands	5
Niche size	2
Migrate	10
Mutate	95
Cross-over	95

Re-docking validation

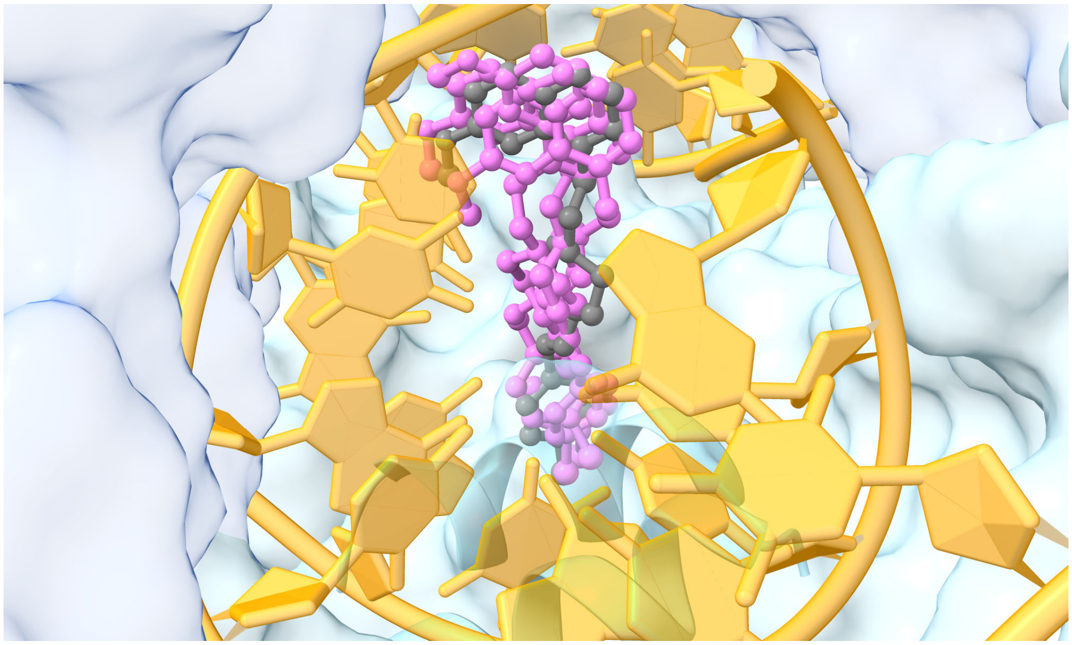

Figure S1. Structural comparison between co-crystallized AMK-12 conformation and its calculated docked poses in the crystal structure of *S. aureus* DNA gyrase (PDB ID: 6Z1A) The co-crystallized ligand AMK-12 is represented in grey, while its re-docked poses in violet.

Table S2. Re-docking validation data

Protein<br>Co-crystallized ligand<br>(PDB ID)	RMDS (Å)		
	Docked pose 1	Docked pose 2	Docked pose 3
S. aureus DNA gyrase<br>AMK-12<br>(6Z1A)	0.832	1.750	1.304

Heavy-atoms root-mean-square deviation (RMSD) values in Ångstrom units (Å) calculated between each AMK-12 re-docked pose and the co-crystallized AMK-12 conformation within *S. aureus* DNA gyrase.

Antimicrobial activity of the NBTIs in μM.

Table S3. Antimicrobial activity of the NBTIs against different Gram-positive and Gram-negative bacteria.

Cmpd	MIC (μM)								
	**5**	**6**	**7**	**8**	**9**	**10**	**11**	**12**	**Gepo**
S. aureus (ATCC 29213)	0.082	2.56	1.28	0.161	164	>328	>328	0.082	0.320
MRSA (QA-11.7) [1]	0.041	2.56	2.56	0.320	ND	ND	ND	0.161	0.161
MRSA(QA-12.1) [2]	0.041	5.12	1.28	0.161	ND	ND	ND	0.082	0.320
MRSA (QA-11.2)	0.082	1.28	0.640	0.082	ND	ND	ND	0.161	1.28
E. coli (ATCC 25922)	5.12	>328	>328	20.5	>328	>328	>328	10.2	2.56
E. coli D22 [3]	2.56	81.9	81.9	2.56	>328	>328	>328	0.640	0.320
E. coli N43 [4]	0.320	10.2	10.2	2.56	164	>328	>328	0.161	0.041
E. coli ESBL QA:11.3	10.2	>328	>328	20.5	>328	>328	>328	10.2	ND
K. pneumoniae	41.0	>328	>328	41.0	>328	>328	>328	41.0	20.5
P. aeruginosa RDK 184	>328	>328	>328	328	>328	>328	>328	>328	20.5
E. faecalis DRK 057	0.320	20.5	10.2	0.640	>328	>328	>328	1.28	5.12
VRE	0.082	5.12	5.12	0.320	ND	ND	ND	0.640	2.56
A. baumannii	2.56	164	164	5.12	>328	>328	>328	5.12	20.5

[1] Resistant to cefoxitin, ciprofloxacin, clindamycin, erythromycin, tetracycline, thiamulin, trimethoprim. [2] Resistant to cefoxitin, gentamicin, kanamycin, rifampicin, streptomycin, sulfamethoxazole, tetracycline. [3] With a mutation in the *lpxC* gene that increases membrane permeability. [4] With *AcrA* knock-out (cell membrane efflux pump). ATCC, American Type Culture Collection; QA, Quality Assurance; Gepo: gepotidacin.

NMR spectra

1-(2-(6-methoxy-1,5-naphthyridin-4-yl)ethyl)-N-(4-methylbenzyl)piperidin-4-amine (5)

^{1}H NMR (400 MHz, $CDCl_3$)

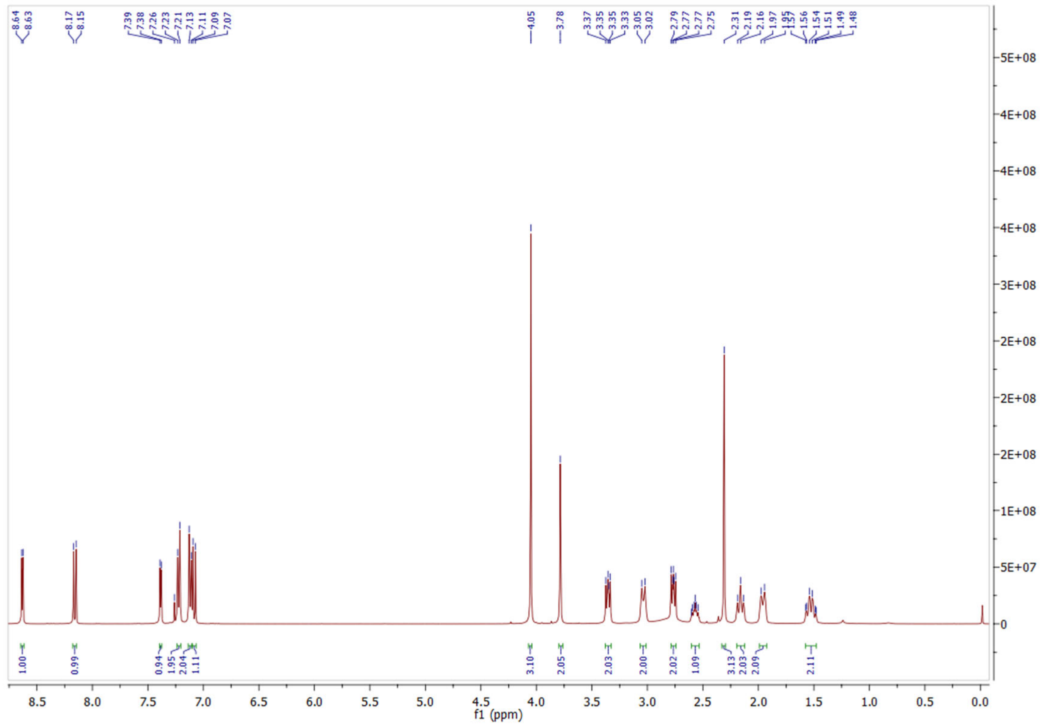

^{13}C NMR (100 MHz, $CDCl_3$)

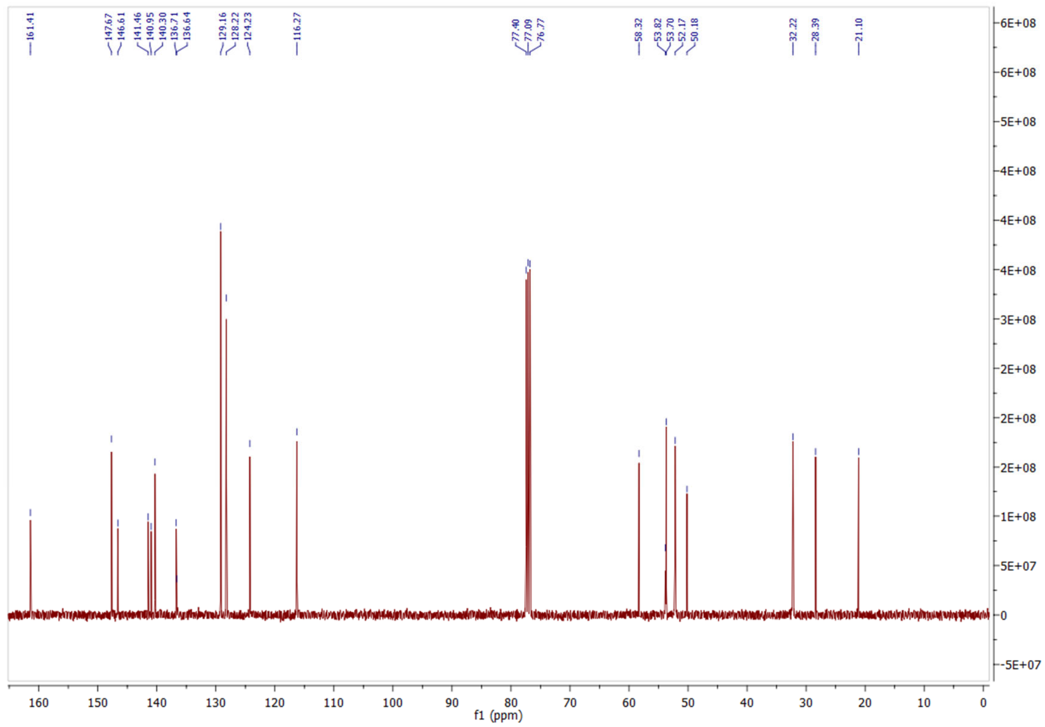

(4-(((6-methoxy-1,5-naphthyridin-4-yl)oxy)methyl)-N-(4-methylbenzyl)cyclohexan-1-amine (6)

^{1}H NMR (400 MHz, $CDCl_3$)

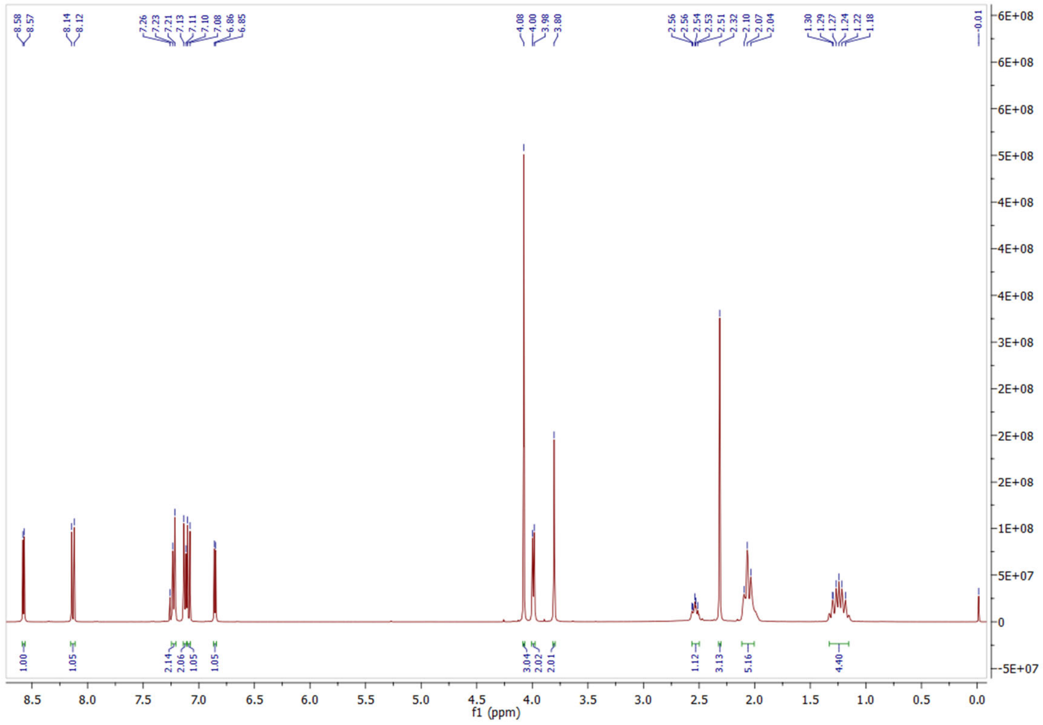

^{13}C NMR (100 MHz, $CDCl_3$)

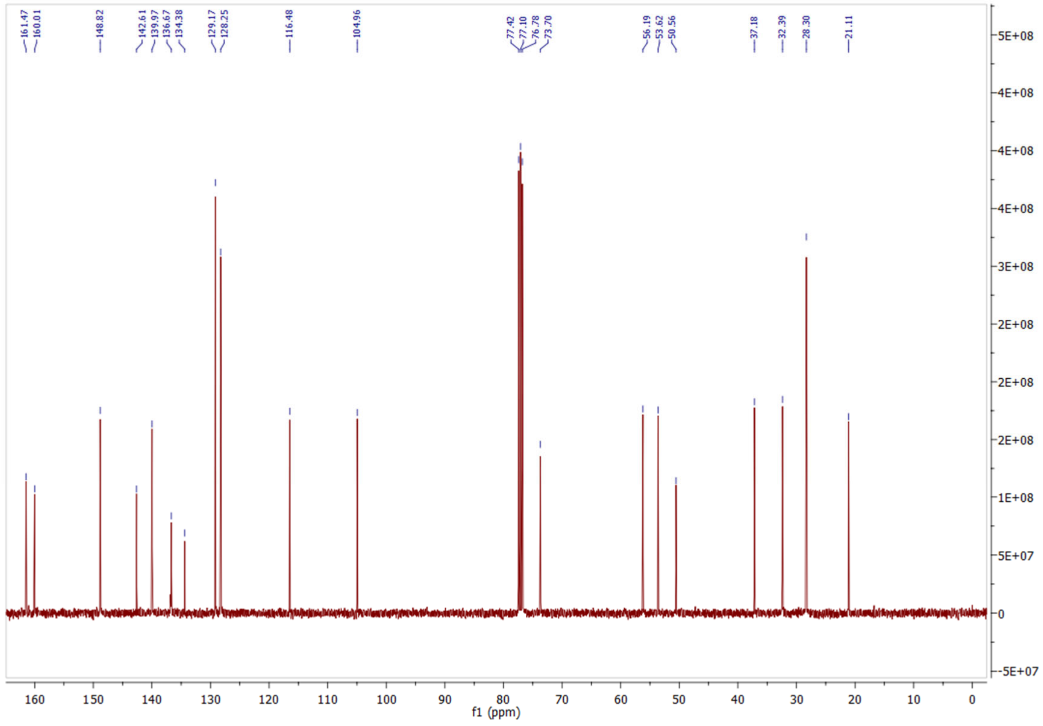

6-(((6-methoxy-1,5-naphthyridin-4-yl)oxy)methyl)-N-(4-methylbenzyl)tetrahydro-2H-pyran-3-amine (7)

^{1}H NMR (400 MHz, DMSO-d_6)

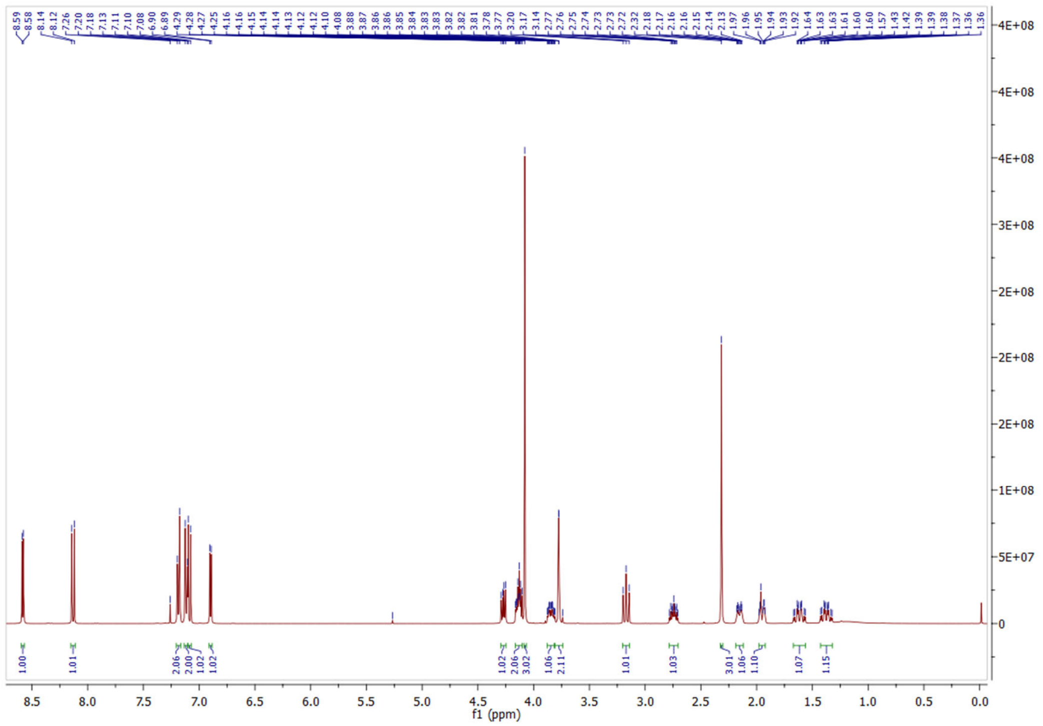

^{13}C NMR (100 MHz, DMSO-d_6)

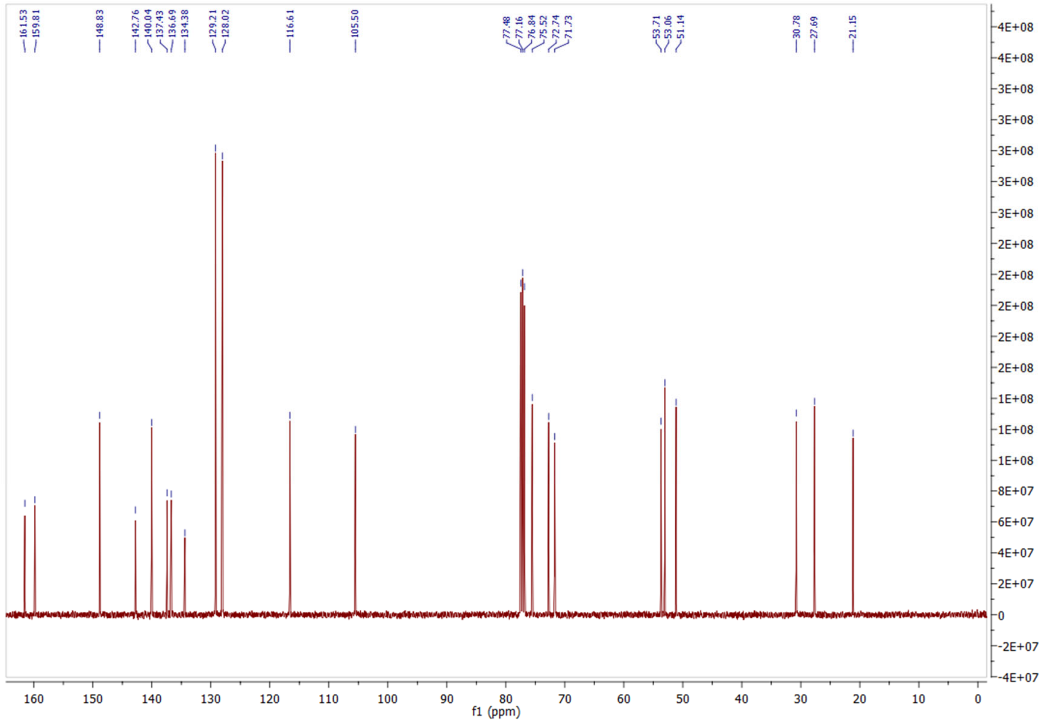

1-(6-methoxy-1,5-naphthyridin-4-yl)-2-(4-((4-methylbenzyl)amino)cyclohexyl)ethan-1-ol (8)

^{1}H NMR (400 MHz, $CDCl_3$)

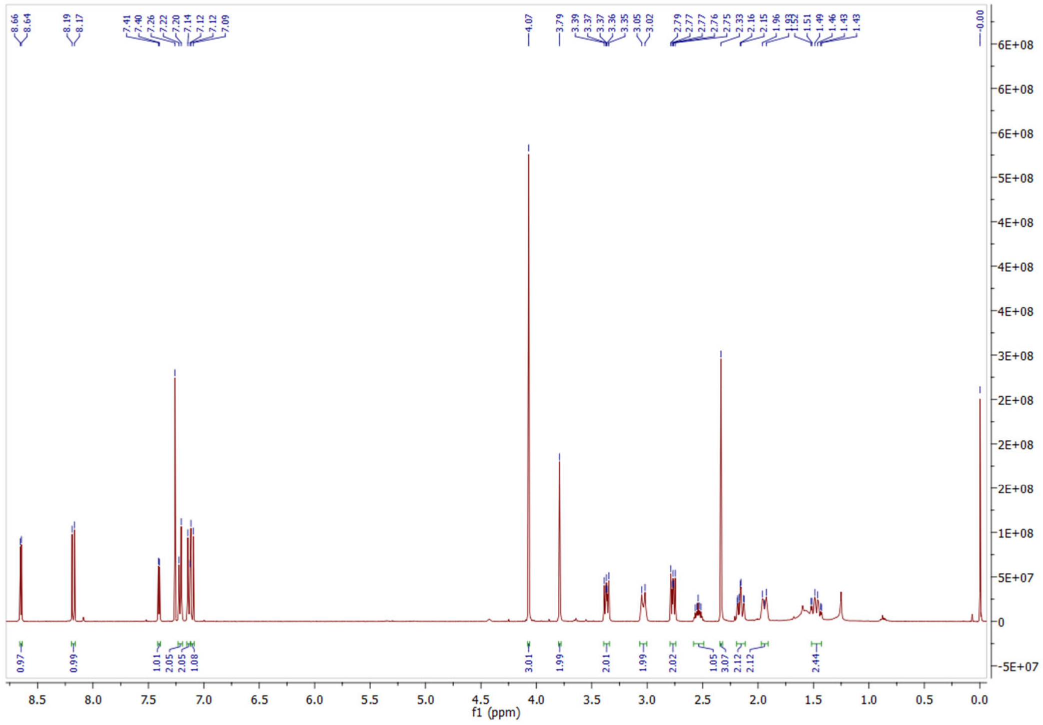

^{13}C NMR (100 MHz, $CDCl_3$)

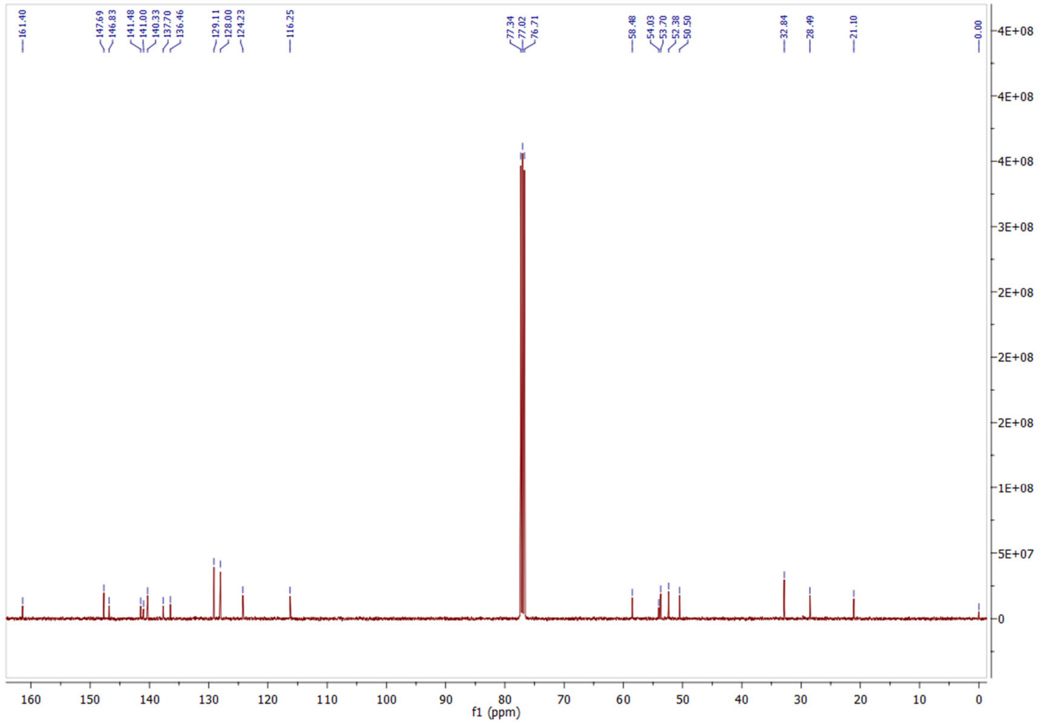

N-(4-aminobenzyl)-1-(2-(6-methoxy-1,5-naphthyridin-4-yl)ethyl)piperidin-4-amine (9)

^{1}H NMR (400 MHz, DMSO-d_6)

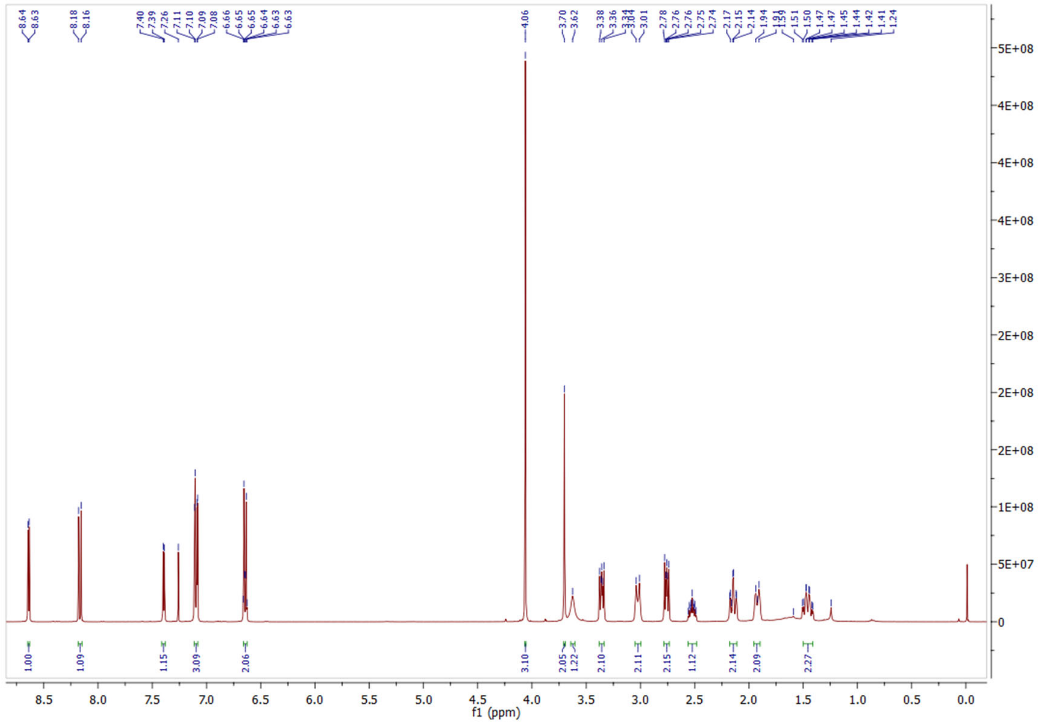

^{13}C NMR (100 MHz, DMSO-d_6)

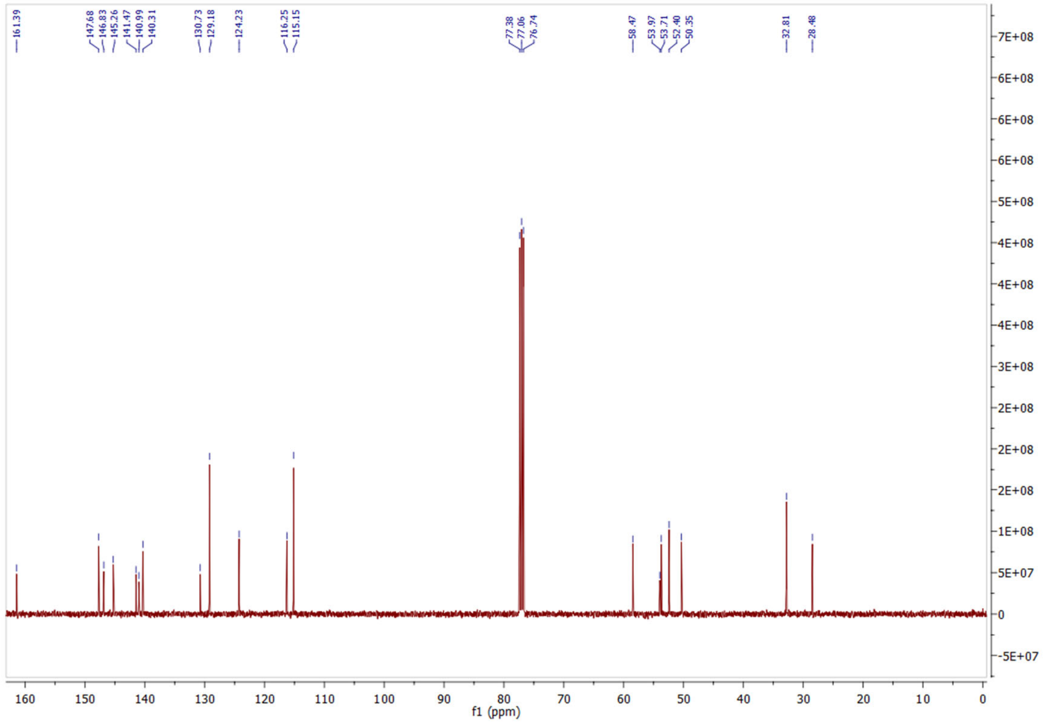

4-(((4-(((6-methoxy-1,5-naphthyridin-4-yl)oxy)methyl)cyclohexyl)amino)methyl)aniline (10)

^{1}H NMR (400 MHz, DMSO-d_6)

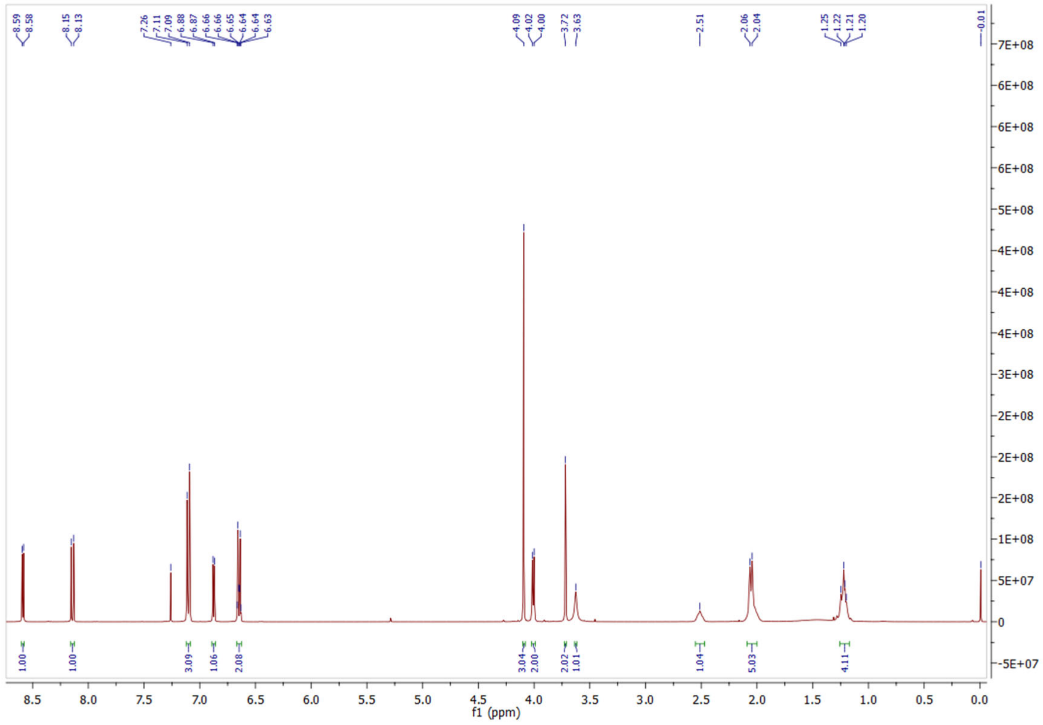

^{13}C NMR (100 MHz, DMSO-d_6)

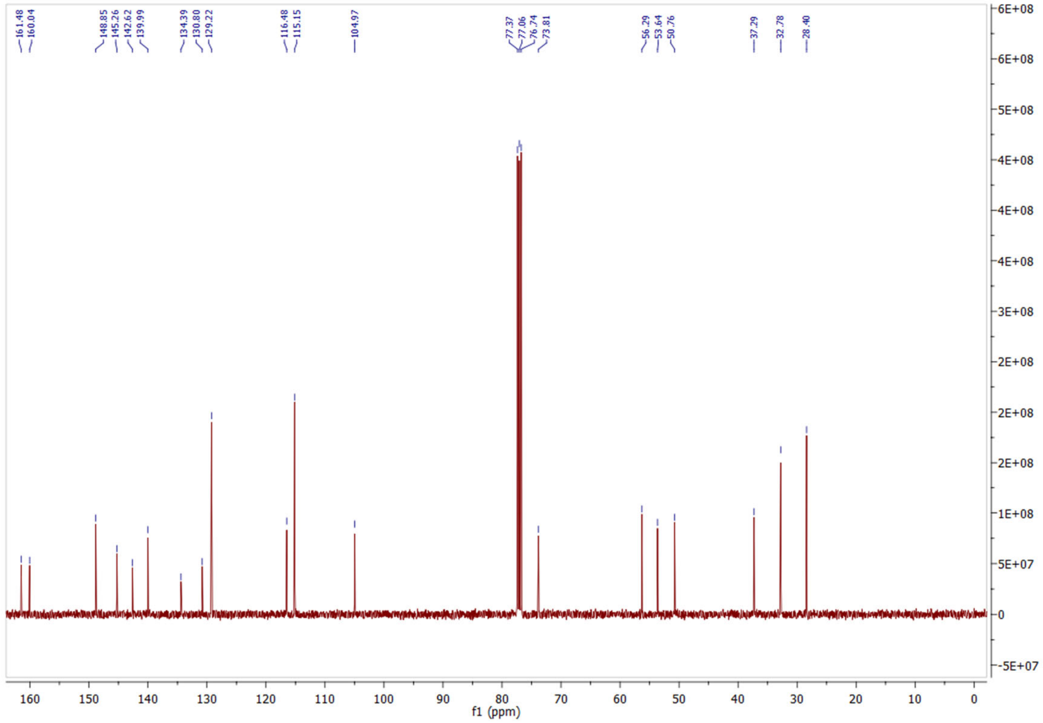

N-(4-aminobenzyl)-6-(((6-methoxy-1,5-naphthyridin-4-yl)oxy)methyl)tetrahydro-2H-pyran-3-amine (11)

^{1}H NMR (400 MHz, DMSO-*d*$_6$)

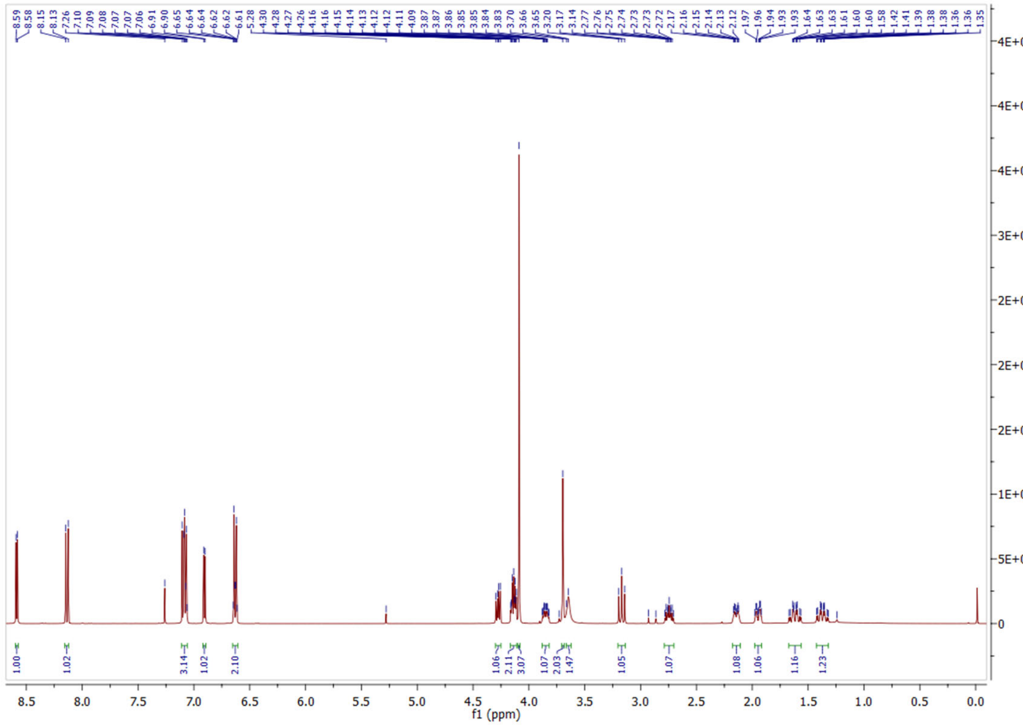

^{13}C NMR (100 MHz, DMSO-*d*$_6$)

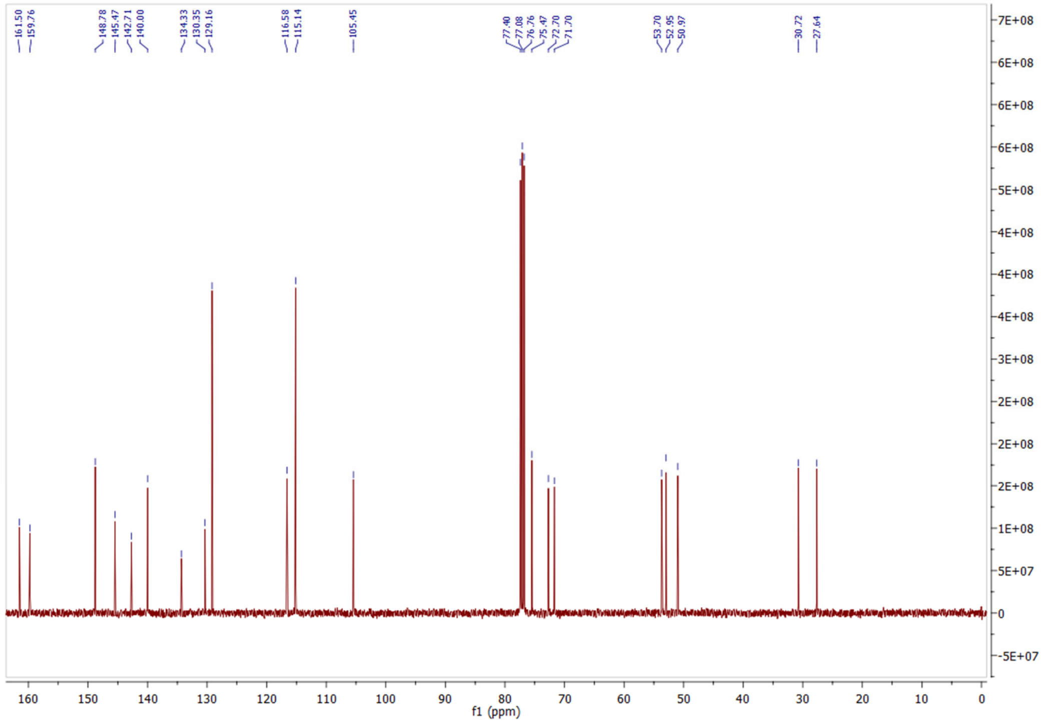

N-(4-bromo-3-(trifluoromethyl)benzyl)-1-(2-(6-methoxy-1,5-naphthyridin-4-yl)ethyl)piperidin-4-amine (12)

^{1}H NMR (400 MHz, DMSO-d_6)

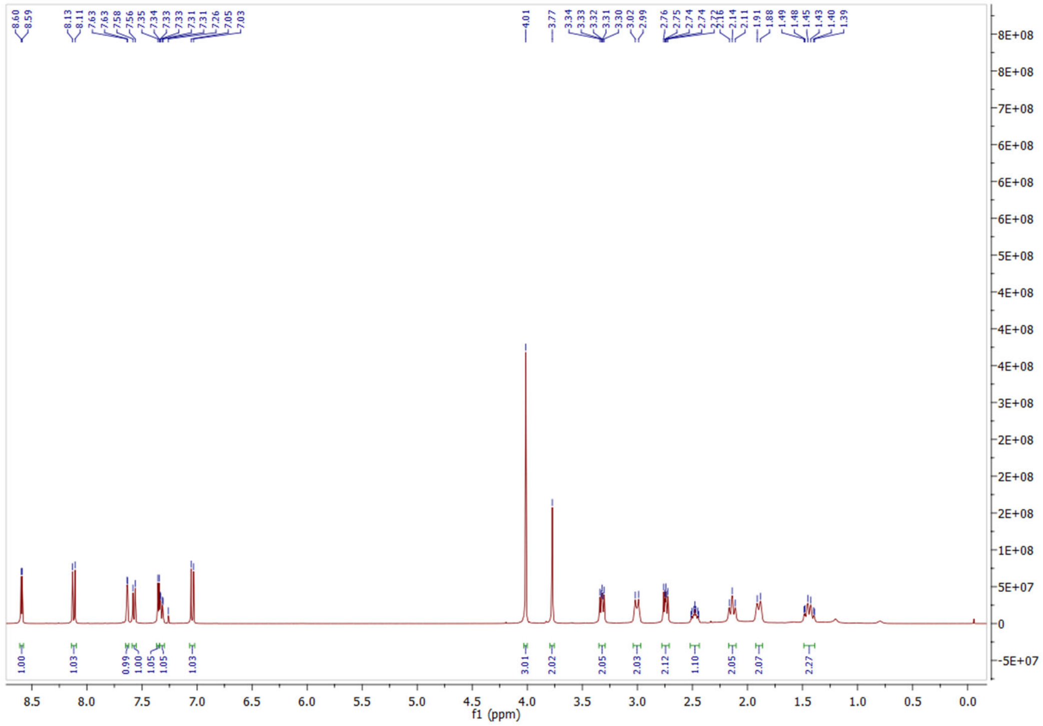

^{13}C NMR (100 MHz, DMSO-d_6)

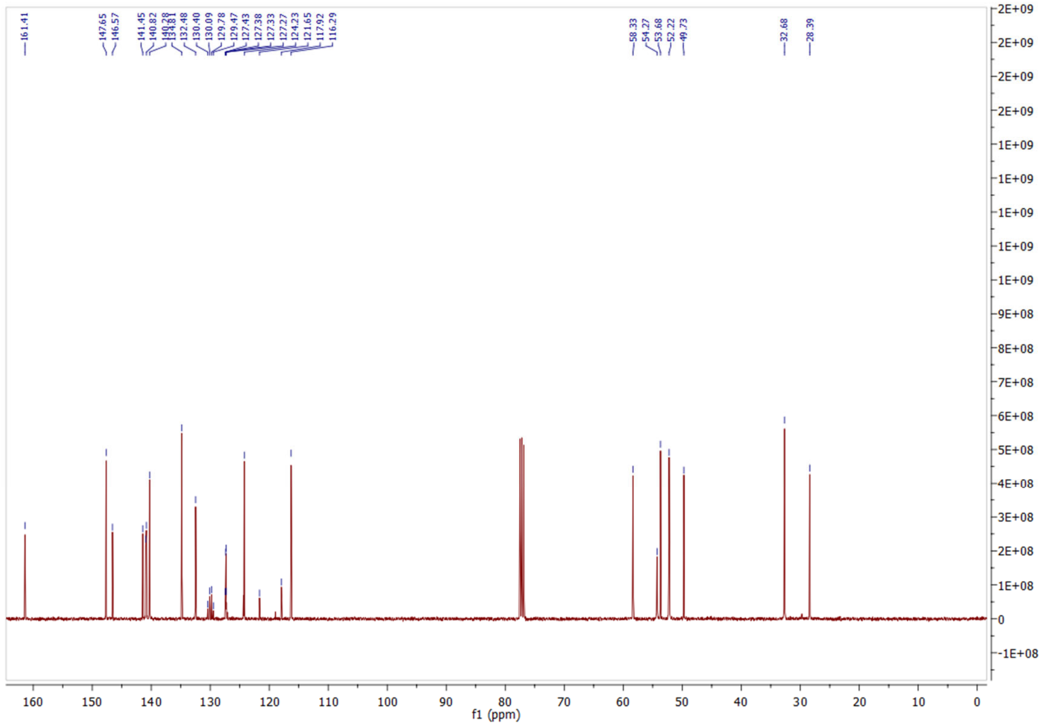

HPLC traces

HPLC chromatogram of hit compound 5 at 254 nm.

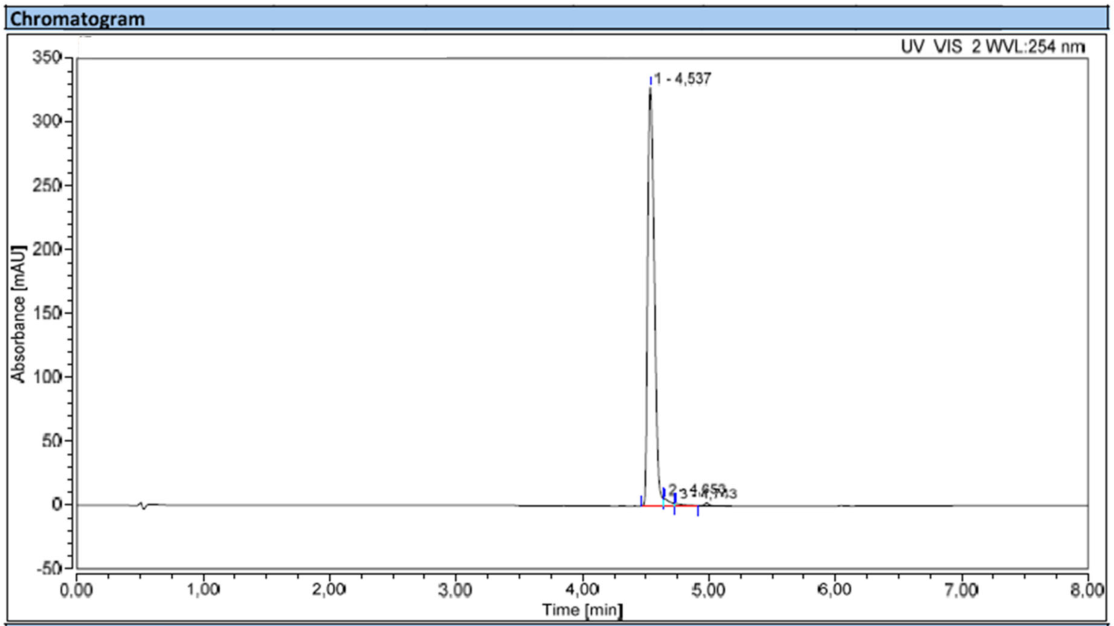

Integration Results

No.	Peak Name	Retention Time min	Area mAU*min	Height mAU	Relative Area %	Relative Height %	Amount n.a.
1		4,537	19,736	327,848	97,45	97,73	n.a.
2		4,653	0,326	5,779	1,61	1,72	n.a.
3		4,743	0,190	1,822	0,94	0,54	n.a.
**Total:**			**20,252**	**335,449**	**100,00**	**100,00**	

HPLC chromatogram of hit compound 6 at 254 nm.

Chromatogram

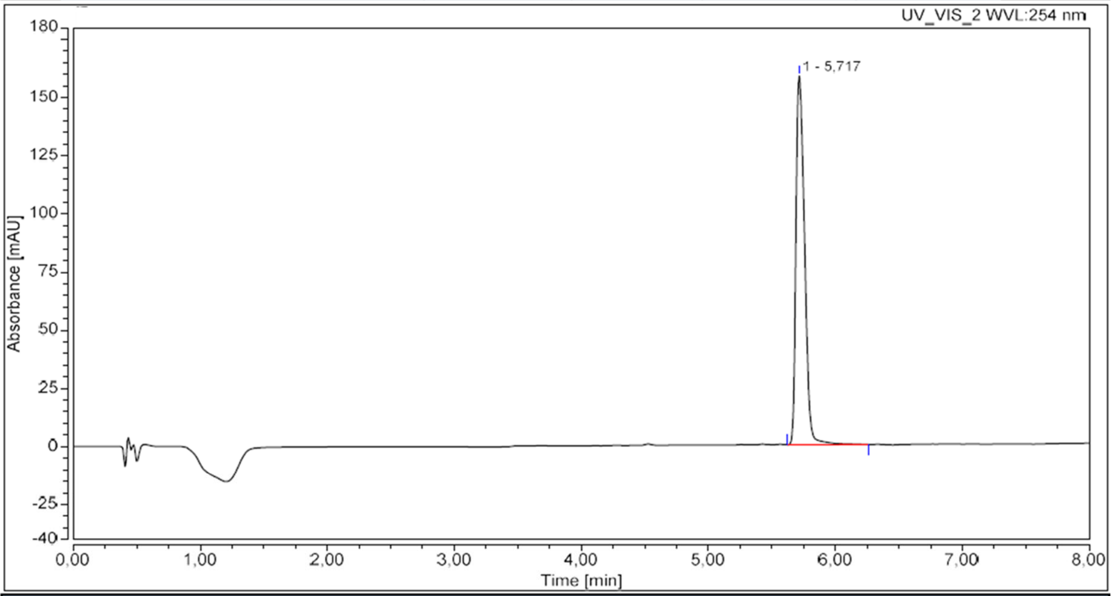

Integration Results

No.	Peak Name	Retention Time min	Area mAU*min	Height mAU	Relative Area %	Relative Height %	Amount n.a.
1		5,717	12,408	158,477	100,00	100,00	n.a.
**Total:**			**12,408**	**158,477**	**100,00**	**100,00**	

HPLC chromatogram of hit compound 7 at 254 nm.

Chromatogram

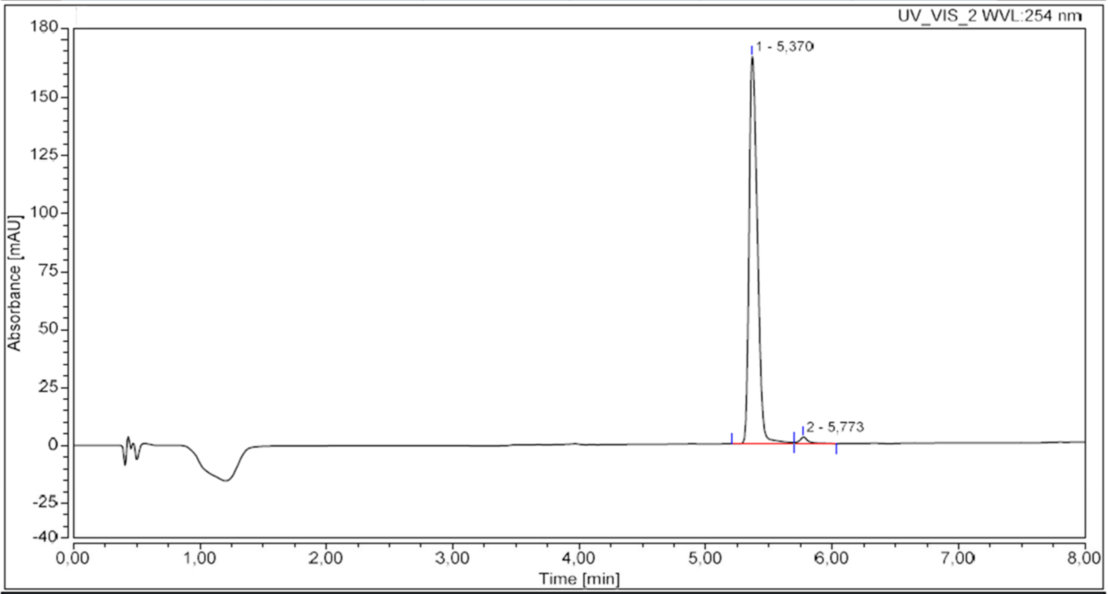

Integration Results

No.	Peak Name	Retention Time min	Area mAU*min	Height mAU	Relative Area %	Relative Height %	Amount n.a.
1		5,370	12,730	167,112	98,12	98,27	n.a.
2		5,773	0,244	2,949	1,88	1,73	n.a.
**Total:**			**12,974**	**170,061**	**100,00**	**100,00**	

HPLC chromatogram of hit compound 8 at 254 nm.

Chromatogram

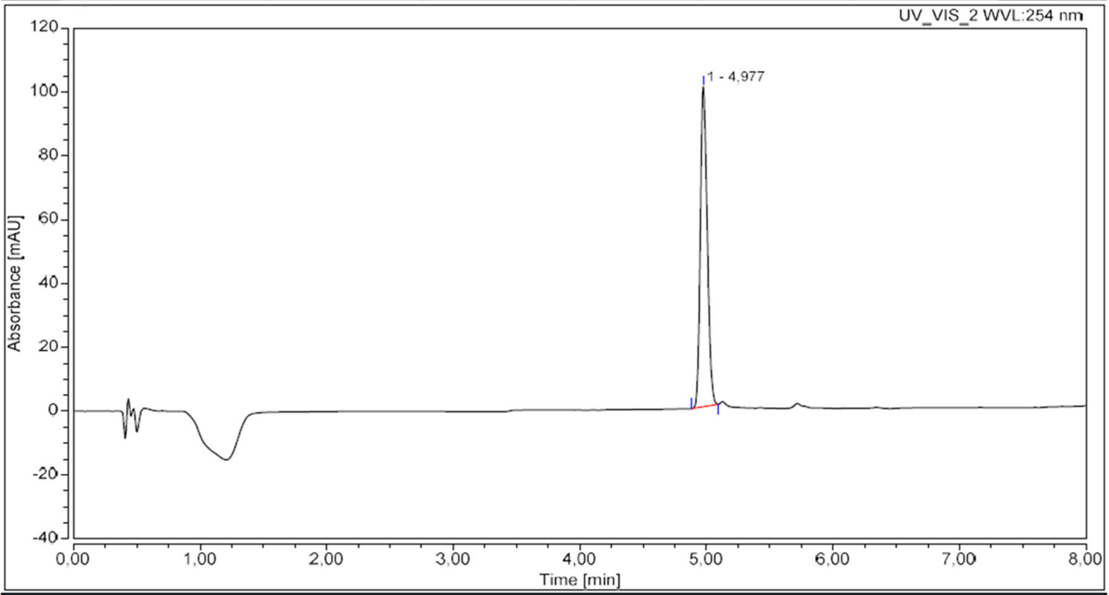

Integration Results

No.	Peak Name	Retention Time min	Area mAU*min	Height mAU	Relative Area %	Relative Height %	Amount n.a.
1		4,977	6,311	100,306	100,00	100,00	n.a.
**Total:**			**6,311**	**100,306**	**100,00**	**100,00**	

HPLC chromatogram of hit compound 9 at 254 nm.

Chromatogram

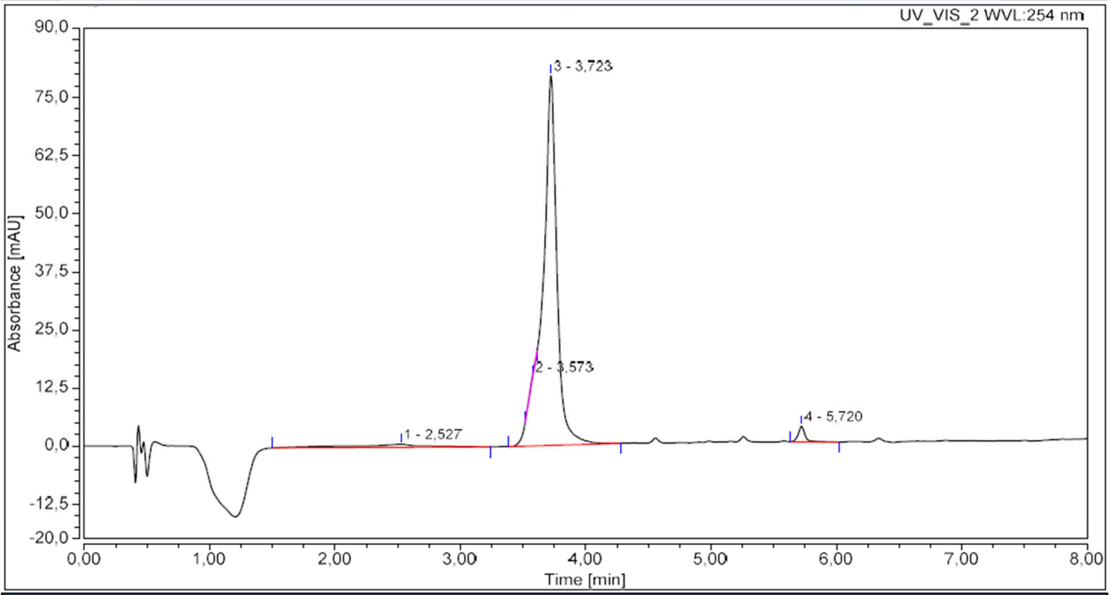

Integration Results

No.	Peak Name	Retention Time min	Area mAU*min	Height mAU	Relative Area %	Relative Height %	Amount n.a.
1		2,527	0,426	0,634	3,73	0,75	n.a.
2		3,573	0,046	0,828	0,40	0,98	n.a.
3		3,723	10,718	79,553	93,95	94,25	n.a.
4		5,720	0,219	3,390	1,92	4,02	n.a.
**Total:**			**11,408**	**84,405**	**100,00**	**100,00**	

HPLC chromatogram of hit compound 10 at 254 nm.

Chromatogram

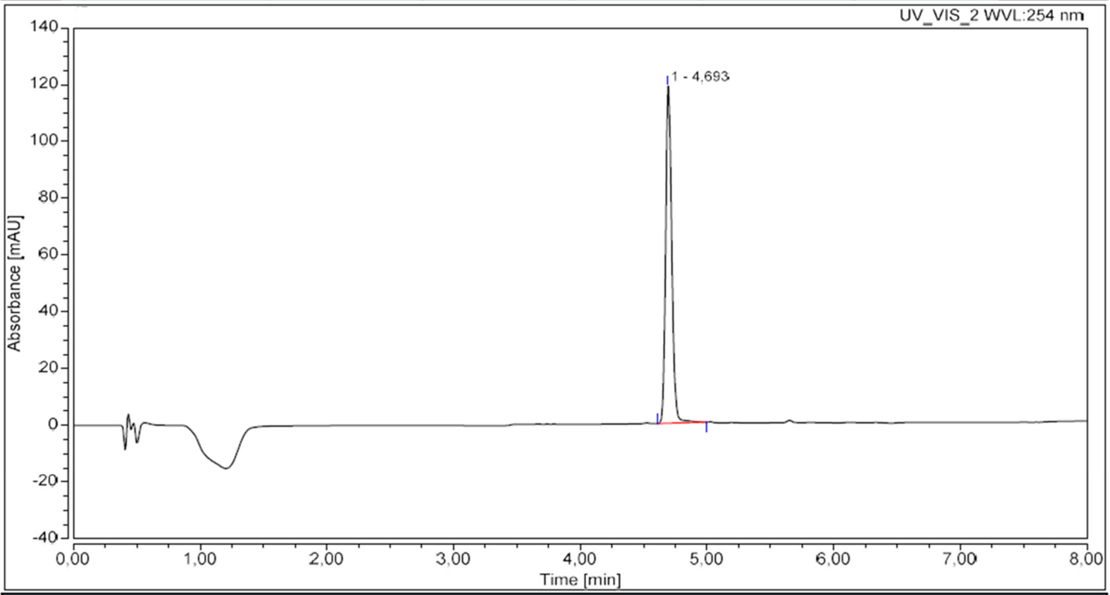

Integration Results

No.	Peak Name	Retention Time min	Area mAU*min	Height mAU	Relative Area %	Relative Height %	Amount n.a.
1		4,693	6,602	118,714	100,00	100,00	n.a.
**Total:**			**6,602**	**118,714**	**100,00**	**100,00**	

HPLC chromatogram of hit compound 11 at 254 nm.

Chromatogram

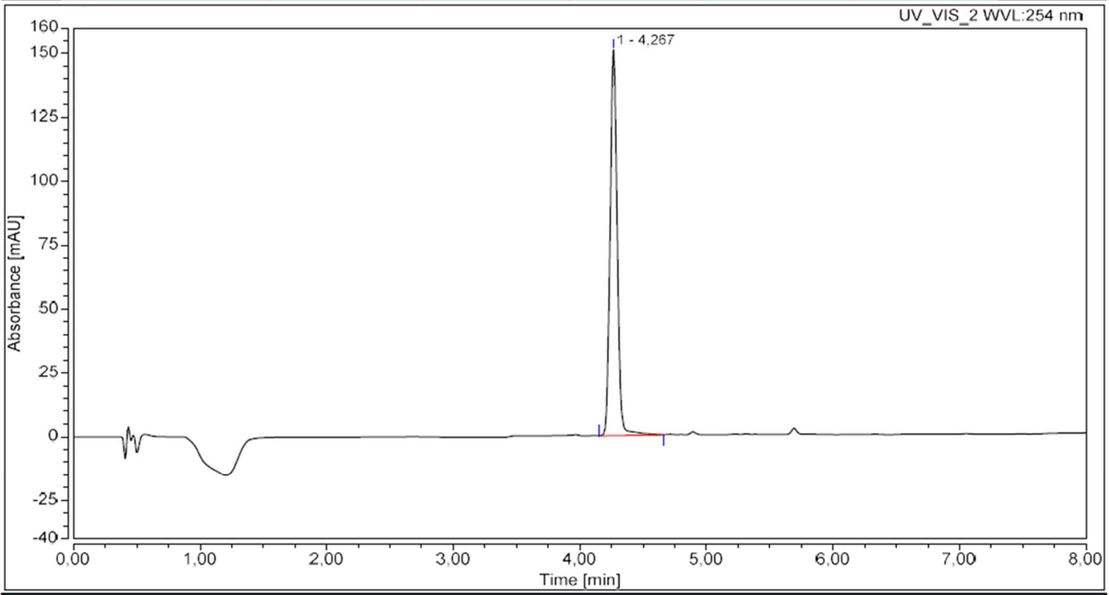

Integration Results

No.	Peak Name	Retention Time min	Area mAU*min	Height mAU	Relative Area %	Relative Height %	Amount n.a.
1		4,267	9,909	151,010	100,00	100,00	n.a.
**Total:**			**9,909**	**151,010**	**100,00**	**100,00**	

HPLC chromatogram of hit compound 12 at 254 nm.

Chromatogram

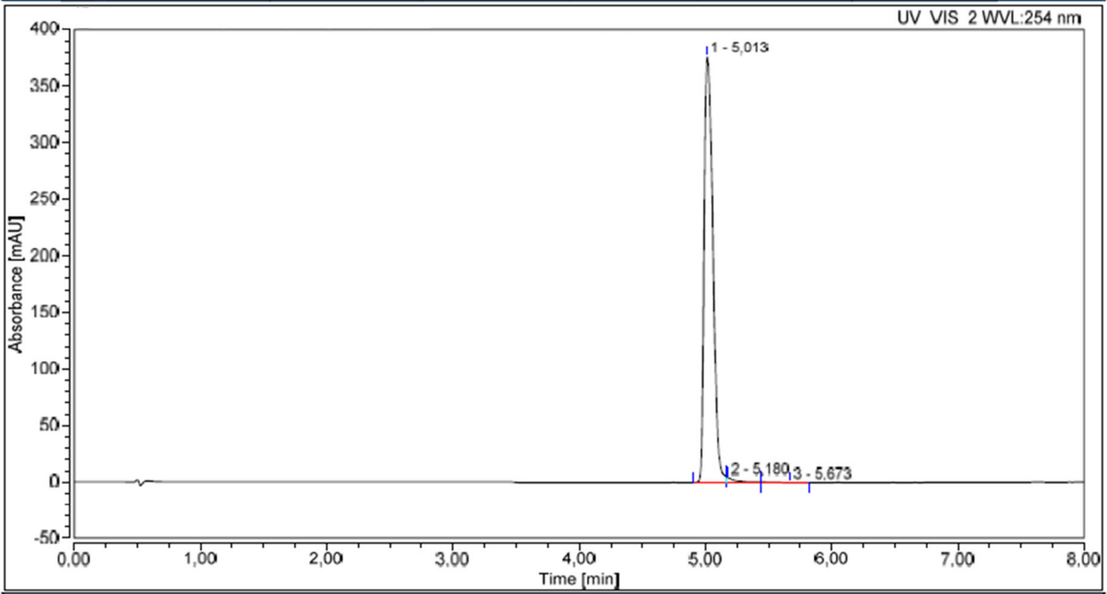

Integration Results

No.	Peak Name	Retention Time min	Area mAU*min	Height mAU	Relative Area %	Relative Height %	Amount n.a.
1		5,013	29,893	376,312	98,42	98,77	n.a.
2		5,180	0,417	4,576	1,37	1,20	n.a.
3		5,673	0,064	0,129	0,21	0,03	n.a.
**Total:**			**30,374**	**381,017**	**100,00**	**100,00**	

HRMS spectra

5

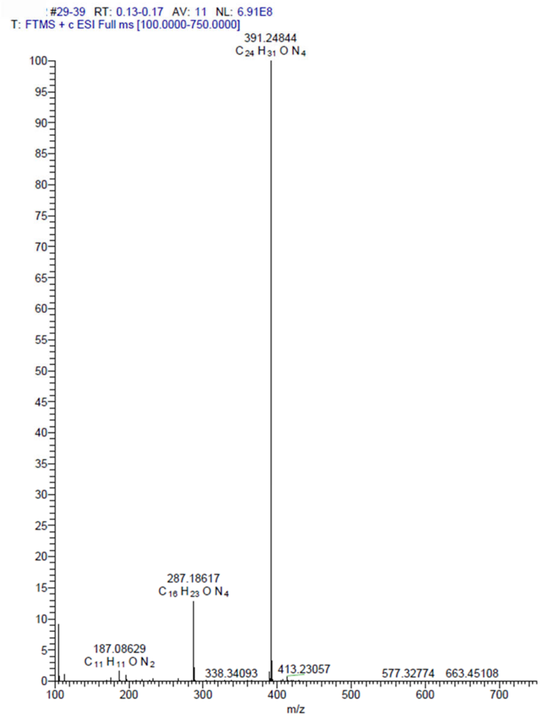

6

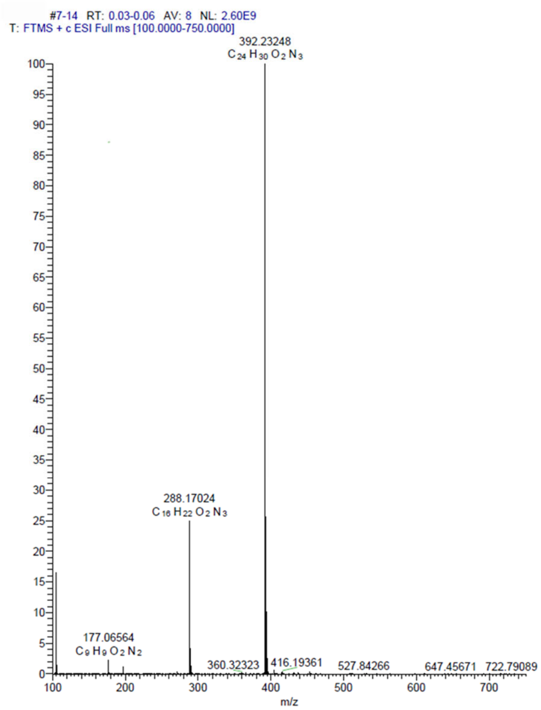

7

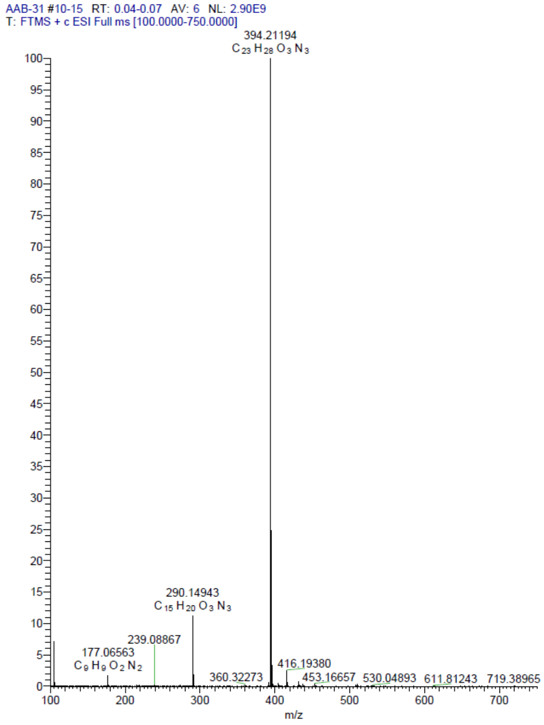

9

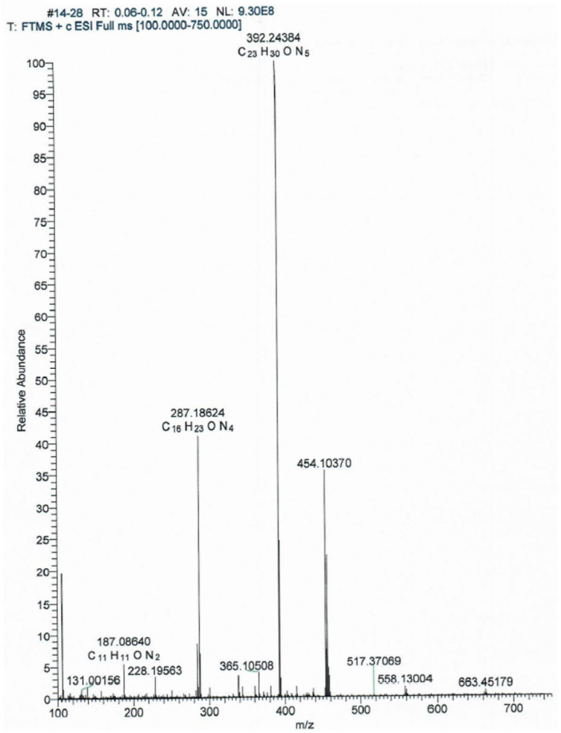

10

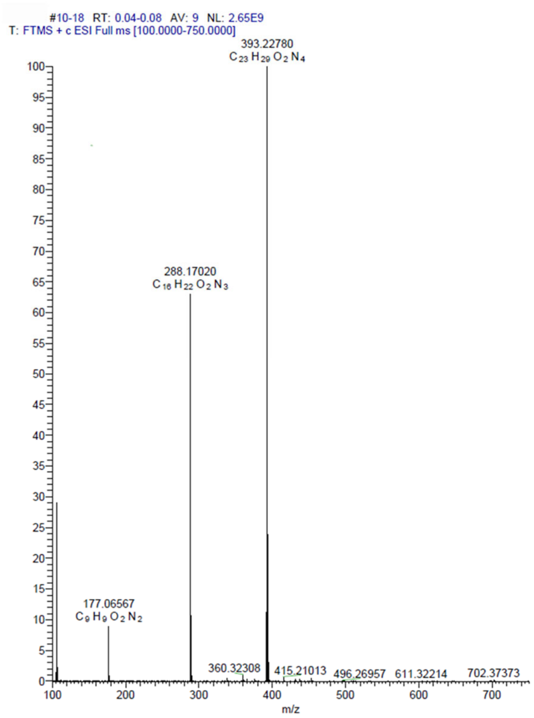
#10-18 RT: 0.04-0.08 AV: 9 NL: 2.65E9
T: FTMS + c ESI Full ms [100.0000-750.0000]
393.22780
C23 H29 O2 N4
288.17020
C16 H22 O2 N3
177.06567
C9 H9 O2 N2
360.32308
415.21013
496.26957
611.32214
702.37373
m/z

11

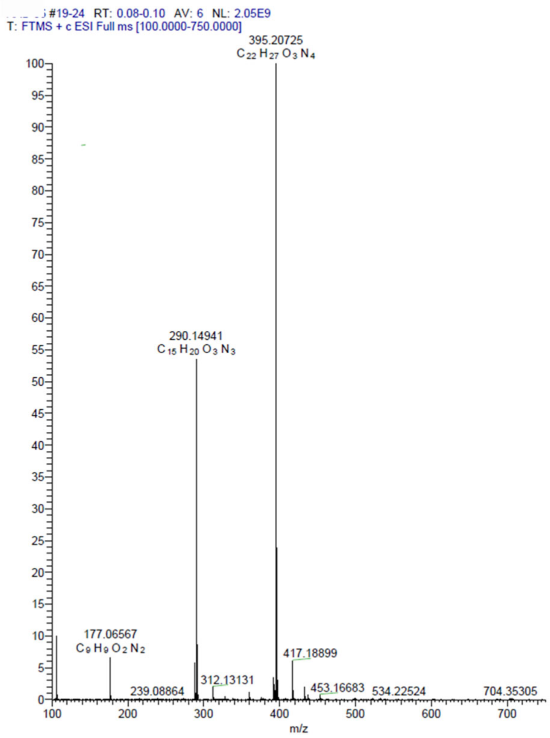
#19-24 RT: 0.08-0.10 AV: 6 NL: 2.05E9
T: FTMS + c ESI Full ms [100.0000-750.0000]
395.20725
C22 H27 O3 N4
290.14941
C15 H20 O3 N3
177.06567
C9 H9 O2 N2
239.08864
312.13131
417.18899
453.16683
534.22524
704.35305
m/z

12

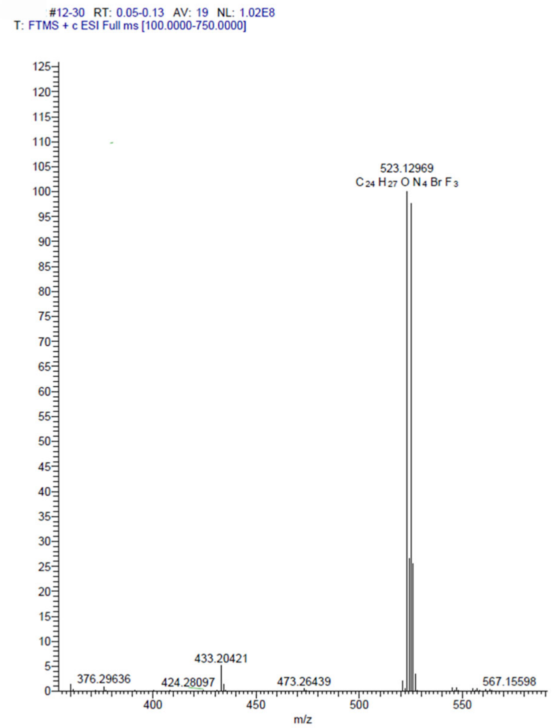
#12-30 RT: 0.05-0.13 AV: 19 NL: 1.02E8
T: FTMS + c ESI Full ms [100.0000-750.0000]
523.12969
C24 H27 O N4 Br F3
376.29636
424.28097
433.20421
473.26439
567.15598
m/z
